# Nudgeability: Mapping Conditions of Susceptibility to Nudge Influence

**DOI:** 10.1177/1745691621995183

**Published:** 2021-08-23

**Authors:** Denise de Ridder, Floor Kroese, Laurens van Gestel

**Affiliations:** Department of Social Health and Organizational Psychology, Utrecht University

**Keywords:** nudges, awareness, preferences, dual-process models

## Abstract

Nudges are behavioral interventions to subtly steer citizens’ choices toward “desirable” options. An important topic of debate concerns the legitimacy of nudging as a policy instrument, and there is a focus on issues relating to nudge transparency, the role of preexisting preferences people may have, and the premise that nudges primarily affect people when they are in “irrational” modes of thinking. Empirical insights into how these factors affect the extent to which people are susceptible to nudge influence (i.e., “nudgeable”) are lacking in the debate. This article introduces the new concept of *nudgeability* and makes a first attempt to synthesize the evidence on when people are responsive to nudges. We find that nudge effects do not hinge on transparency or modes of thinking but that personal preferences moderate effects such that people cannot be nudged into something they do not want. We conclude that, in view of these findings, concerns about nudging legitimacy should be softened and that future research should attend to these and other conditions of nudgeability.

Since its introduction more than a decade ago, nudging as a policy instrument to steer citizen choices in a gentle way has been met with great enthusiasm and extensive criticism. The enthusiasm has been most prominently observed among policymakers, who were eager to apply psychological insights to promote behavior change after having witnessed the limited success of educational public campaigns (Dolan et al., 2012). The positive vibe was further fueled by reported successes of nudge interventions in a variety of public-policy cases in which people’s choices are critical, such as pension schemes, sustainable actions, and health behavior (Benartzi et al., 2017). Alongside these optimistic adoptions of nudging in public policy, however, its use as a policy instrument has been heavily debated by scholars, politicians, and members of the public (Grüne-Yanoff, 2012; House of Lords, Science and Technology Select Committee, 2011; McCrudden & King, 2016; Wilkinson, 2013).

The core of debates on nudging has been the question of legitimacy: Is it admissible to subtly guide people’s behavior in the desired direction? More specifically, who determines what behavior is “desired,” and what if individuals have other ideas about the desirability of certain choices than policymakers or other choice architects? Whereas some have suggested that many citizens would probably “thank public officers for making the choice easy for them” (John, 2018, p. 110), others have warned that governments considering nudging as a policy instrument should be aware that appropriate democratic control procedures are essential for establishing common ground for the presumed desirability of one choice over others (Button, 2018; Lepenies et al., 2018). Another point of discussion has been the “subtle guidance”: The subtleness of nudging interventions gives rise to concerns of manipulation and even indoctrination, leading people to certain choices outside of their control and thereby infringing on their autonomy. Yet others have argued that nudging may actually enhance autonomy to the extent that it facilitates the choice that individuals would have made given the opportunity (Saghai, 2013; Vugts et al., 2018).

Whatever stance they may have, both parties’ opinions on the legitimacy of nudging as a policy instrument are rooted in assumptions about how nudges operate on a psychological level. Supporters and critical followers alike endorse the premise that nudges easily influence behavior and produce straightforward predictable effects on decision-making because they speak to “fast” nonreflective System 1 reasoning, which is characterized by the absence of deliberation (Marchiori et al., 2017; see Box 1). Moreover, both sides agree on the notion that nudges are effective because people are unaware of their presence and purpose (Hansen & Jespersen, 2013; Smith et al., 2013). Proponents and critics also concur with the idea that nudges have an impact on choice regardless of preexisting preferences for a specific option—which is a major advantage for some (Martin et al., 2014; Service et al., 2014) and an issue of concern for others (John et al., 2009).

Box 1 **System 1 and System 2 Decision-Making**

The theoretical principles on which nudges are based have been known since the introduction of dual-processing accounts of human behavior in the 1970s. Although there is a plethora of different dual-processing theories (Evans, 2008), they all share the distinction between two distinct modes of processing, generally labeled System 1 and System 2. System 1 is typically characterized as automatic, fast, and effortless, whereas System 2 is described as controlled, slow, and effortful. System 1 processes generally occur with few cognitive investments, whereas System 2 is thought to tax working memory capacity. The most frequently used model to describe how these systems operate is the default-interventionist model, which describes System 1 processing as the default mode and System 2 as the mode that intervenes if necessary. System 1 processes were originally characterized as suboptimal and as leading to erroneous judgments. However, System 1 is no longer seen as responsible for errors and biases but is seen as adaptive (Gigerenzer, 2015). Moreover, recent refinements of the dual-processing theories has led to consensus of two types of reasoning with typical correlates—as opposed to two systems (Bago & De Neys, 2019; Melnikoff & Bargh, 2018). Although we acknowledge these recent advances, for ease of interpretation we cluster the typical correlates of the two types of reasoning under the umbrella terms of System 1 and System 2.

However, the validity of these assumptions has yet to be determined. Accumulating evidence suggests that choices are not as easily modified by nudging as generally believed (Gigerenzer, 2015), with several systematic reviews (Diepeveen et al., 2013; Szaszi et al., 2018) and meta-analyses (Cadario & Chandon, 2019; Hollands et al., 2013; Hummel & Maedche, 2019) demonstrating that nudging effects are relatively modest regardless of the nudge type and/or target behavior. These documented deviations from what the prevailing understanding of nudge effectiveness would predict call for further scrutiny of the conditions supposedly determining the impact of nudges on people’s choices. In this article, we examine the empirical evidence on these conditions, which we label as elements of *nudgeability*, or susceptibility to being affected by a nudge. Nudgeability is a new concept that we introduce to refer to conditions that determine to what extent people are receptive to the influence of nudges. By highlighting these conditions we aim to shift the discussion from straightforward effectiveness toward the more pressing questions of when and why nudges may influence choices. This shift is important for the ongoing debate on the legitimacy of nudging as a policy instrument; nudge critics express concern that nudges may thwart preferences and steer decisions into directions people are unaware of and nudge proponents stating that nudges are always favorable. These kind of strong views, whether for or against nudging, are not very helpful in moving the discussion forward (de Ridder et al., 2020). A more nuanced understanding of the impact of nudges on choice may redirect this debate and soften concerns about the appropriateness of psychological interventions as a policy instrument. By introducing the concept of nudgeability, we do not intend to give another overview of nudge effectiveness. Rather, our aim is to rethink when and why people can be nudged into desirable choices.

Although a wide range of factors may determine when or whether people are likely to be influenced by a nudge, the topic has typically been underinvestigated, and a systematic grouping of relevant features is lacking. Nevertheless, the issues that are central in the debate about the legitimacy of nudging as a policy instrument offer a good point of departure for mapping these factors. We therefore focus on these issues for reviewing nudgeability conditions. Specifically, we discuss (a) the extent to which people are aware of the presence and/or purpose of a nudge either or not in conjunction with explicit warnings of a nudge being in place; (b) people’s preexisting preferences for a specific behavior as witnessed by their personal goals, intentions, and/or motivation to engage in the behavior that is targeted by the nudge; and (c) their modus of thinking, that is, either being inclined to fast System 1 reasoning or slow System 2 deliberating.

We also propose some topics that deserve more attention in future studies to improve our understanding of which conditions make people more (or less) susceptible to the influence of nudges. These conditions relate to, for example, the source of the nudge (government or other parties), the topic of the nudge (relatively smallish issues or topics of heated societal debate), and people’s living conditions (privileged or underprivileged). Despite their relevance, the scarce literature precludes a thorough investigation of how these latter factors affect nudgeability. This especially applies to the important question of whether people from disadvantaged groups are more or less susceptible to nudge influence, as concerns have been raised about nudges potentially increasing the socioeconomic-status (SES) gap in welfare (Ghesla et al., 2020), whereas others have argued that low-SES groups may especially benefit from nudge interventions (Marteau et al., 2012).

It should be emphasized that the conditions that determine whether people are receptive to nudge influence have not yet been the topic of systematic analysis. This article is therefore not meant to be exhaustive but rather a first attempt at categorizing scattered findings. Our previous studies on transparency (Wachner et al., 2020a, 2020b), preferences (Venema, 2020; Venema et al., 2019, 2020), and modes of thinking (Van Gestel et al., 2020) have been leading in generating a body of evidence on the work that has been done in these areas. Related studies examined in the context of this research served as a starting point for the current article, which explores these findings from the viewpoint of nudgeability. The relatively limited number of studies on the moderators of nudge effectiveness that we discuss in this article corroborates the conclusion of a recent systematic review stating that only 24% of 422 nudge interventions explored boundary conditions or underlying mechanisms of effectiveness (Szaszi et al., 2018). We document what is known about nudgeability without pretending that it covers all studies that could be relevant (e.g., we did not search for unpublished studies or gray literature, which would be a significant next step in charting out nudgeability).

In doing so, we do not target specific categories of nudges but rather use the generic term “nudge” to describe a range of interventions that may differ in scope and design but have in common the aim to gently steer a choice without forbidding the alternative option (Thaler & Sunstein, 2008). We are aware that different categorizations of nudges have been proposed (e.g., Dolan et al., 2012; Hollands et al., 2017; Münscher et al., 2016). However, to date there is no widely accepted grouping of nudge interventions (Marchiori et al., 2017), which precludes a systematic investigation of nudgeability depending on a particular type of nudge. Having said this, we acknowledge that different nudges may speak to different psychological mechanisms. Although nudges are generally regarded as typical System 1 devices that aim to target fast automatic thinking (Thaler & Sunstein, 2008), another type of nudges has been introduced that would appeal to more reflective decision-making, such as System 2 or “educational” nudges (Sunstein, 2016). Insofar as different types of nudges may speak to different ways of reasoning, we discuss these differences in terms of being susceptible to (any kind of) nudge resulting from the mode of information processing (i.e., System 1 or 2) rather than as a characteristic of nudges.

## Transparency and Awareness

It is often stated that nudges work only “in the dark” (Bovens, 2009) and that effectiveness typically depends on people not being aware that their choice is being influenced (Hansen & Jespersen, 2013; Smith et al., 2013; Steffel et al., 2016). This notion may originate from the central premise of nudge theory that nudges target automatic “nonconscious” processes. Indeed, it has been observed that many people who are being nudged do not spontaneously notice the presence of a nudge (Hunter et al., 2018; Kroese et al., 2015; Van Gestel et al., 2018). The idea that unawareness is a critical condition for the impact of nudges may also relate to the impression that many people are reluctant to patronizing directions of their choice, especially insofar as governmental guidance is concerned (Schroeder et al., 2017). This notion asserts that alerting people about the presence of a nudge would make them feel that they were being pushed toward a specific choice, leading to reactance (Wortman & Brehm, 1975) that would then reduce or eliminate the influence of a nudge. Transparency is thus an essential element in discussions on nudge effectiveness. Note that by using the term “transparency” we refer to attempts that make individuals aware of the presence and purpose of a nudge to enable them to recognize how and why a nudge would affect their choice (Loewenstein et al., 2014). This is a more stringent requirement than the public-policy interpretation of nudge transparency, necessitating governments to implement only those policies that they would be willing and able to defend publicly to citizens (Thaler & Sunstein, 2008).

Several studies have demonstrated that disclosure of the presence of a nudge (Bang et al., 2018; Cheung et al., 2019; Kroese et al., 2015; Loewenstein et al., 2015), its purpose (Bruns et al., 2018; Steffel et al., 2016), or the way it works (Bruns et al., 2018; Steffel et al., 2016) does not significantly lower its impact compared with a condition in which disclosure was absent, regardless of whether disclosure was given before or after nudge exposure (Loewenstein et al., 2015). See Box 2 for examples of transparent default nudges. Disclosure of the purpose of the nudge and/or explanation of its potential influence also did not reduce effectiveness in people who were more inclined to be opposed to choice direction (i.e., high in psychological reactance; Bruns et al., 2018). One study showed that the impact of a nudge was even greater when its presence and purpose were revealed; twice as many participants stayed with the default (a preselected option for the duration of online study participation) after having been informed that a nudge was in place (Paunov et al., 2018, Study 3; see also Paunov et al., 2019). According to the authors, this enhanced effect may result from nudgees feeling less deceived because of the disclosure. It has also been suggested that disclosure does not compromise default effects because it may lead to a better understanding of how nudges operate. People who had the opportunity to experience the influence of a nudge (which is considered a superior form of disclosure) believed that it would be more effective than when the effect was simply explained to them in a verbal description (Bang et al., 2018).

Box 2 **Examples of Transparent Defaults**

Several studies have focused on the role of transparency for nudges’ effectiveness. Most of these studies have been conducted with defaults presenting a preferred choice as the standard option. As a choice architect, one can be transparent about the presence of the nudge (disclosing that one option has been preselected), the source of the nudge (revealing the person or organization issuing the nudge), the purpose of the nudge, or the mechanism of the nudge (explaining the way the nudge is supposed to work). Disclosure of this information often comes in the form of written information. Typical examples include:

“Please consider that the preselected default values might have an influence on your decision” (information about the presence of the default; Bruns et al., 2018).

“Please note that you can change the preselected electives to other alternatives. In order to do so, you can visit the administration department and file a change form” (information about the source of the default; Paunov et al., 2018).

“Please consider that the preselected default is meant to encourage higher contributions for the climate protection fund” (information about the purpose of the default; Bruns, 2018).

“Please note the following: We know that in decision situations, people often stick with a choice option which is preselected for them. Therefore we have preselected [option X]” (information about the mechanism of the default; Paunov et al., 2019).

It should be noted that most disclosure studies (Bruns et al., 2018; Loewenstein et al., 2015; Paunov et al., 2018; Steffel et al., 2016) have been conducted with defaults, which are considered to be the most powerful type of nudge (e.g., Jachimowicz et al., 2019; Madrian & Shea, 2001). Although popular in public policies concerning organ donation (Johnson & Goldstein, 2003), pension schemes (Thaler & Benartzi, 2004), and green energy (Allcott & Mullainathan, 2010), default nudges are not uncontroversial because their effectiveness may depend on deceit (House of Lords, Science and Technology Select Committee, 2011). That is, more than other types of nudges, default effects are presumed to rely on people being unaware of their presence and purpose. However, the very finding that default effects persist despite disclosure seems to indicate that people may be more aware of a default being present than is sometimes assumed. Indeed, it has been suggested that people may perceive defaults as implicit or inferred expert recommendations (Johnson et al., 2012; McKenzie et al., 2006; Steffel et al., 2016). This interpretation is supported by a series of experiments in which disclosure was given either with or without revealing the nudging agent, which showed that the default (preselection of elective courses in a hypothetical scenario) was more effective when the source of the nudge (the university administration) was revealed (Paunov et al., 2018, Studies 1 and 2).

However, except for the studies by Paunov and colleagues (2018), the source of the nudge so far has been studied only in relation to nudge acceptability (rather than effectiveness). For example, it has been shown that nudges designed by researchers are better trusted than nudges issued by government (Osman et al., 2018). Similar findings were reported in a study by Junghans et al. (Junghans et al., 2015; see also Evers et al., 2018), in which people reported more trust in nudges implemented by private parties than nudges issued by government. Notwithstanding these findings, a recent study suggested that the effects of the source may be negligible because acceptability is unequivocally high regardless of whether the nudge was implemented by researchers, government, or advertisers (Gold et al., 2020). Rather than the source, it may be the intention of the source that predicts nudge acceptability (Bang et al., 2018).

The need for transparency has been mainly defended from the perspective that nontransparent nudges would hurt the decision maker’s autonomy. This claim, however, is not supported by empirical data on autonomy in relation to nudging but rather is based on perceptions and expectations regarding nudging and decision-making. A recent study found that participants who were exposed to a hypothetical nudge scenario expected that nudges would harm their autonomy (Wachner et al., 2020a). However, when another group of participants actually encountered the same nudge, they did not report lower autonomy (Wachner et al., 2020b). Indeed, it has been suggested that hypothetical-choice scenarios may be an underestimation of nudge appreciation because people tend to be more critical when imagining how nudges would influence their choice than when having actually experienced its influence (Bang et al., 2018). This finding corresponds with a considerable amount of research concluding that people are bad forecasters and perform poorly in predicting emotional responses (Gilbert & Wilson, 2000), the likelihood of specific events to occur (Wilson et al., 2000), or their reactions to future events (Buehler & McFarland, 2001). With these findings in mind, it is important to note that most studies on transparency involved hypothetical scenarios (Bang et al., 2018; Bruns et al., 2018; Loewenstein et al., 2015; Paunov et al., 2018; Steffel et al., 2016) that may bear little relevance to how disclosure affects the effectiveness and appreciation of nudges involving real-life decisions.

Together, these findings suggest that nudge effects persist despite disclosure. In view of generally favorable opinions about nudging in the general public, both in hypothetical scenarios (Diepeveen et al., 2013; Evers et al., 2018; Junghans et al., 2015; Reisch & Sunstein, 2016; Sunstein et al., 2018) and real-life settings (Kroese et al., 2015), it may be not surprising that nudge transparency does not eradicate effects on choice. This implies that the presumed trade-off between legitimacy (requiring transparency about the presence of a nudge) and effectiveness (allegedly precluding transparency) may be much smaller than has been suggested (Bovens, 2009).

## Preexisting Preferences

Related to concerns about nudges “operating in the dark,” critics fear that nudge interventions would manipulate people into making choices they would otherwise never endorse. Nudge proponents have typically argued that such interventions are “asymmetrically paternalistic” (Camerer et al., 2003) in the sense that they would only benefit but never hurt people: When people cannot make deliberated choices, nudge interventions will guide them toward choices that are in their best interest while causing no harm to rational decision makers. The crucial question, however, is whether nudges can guide people toward choices they would not have made had they deliberated their decision. This is particularly relevant in cases in which it is not obvious which decision would be in people’s “best interest” (Goldin, 2015). Nudges should theoretically preserve freedom of choice (Thaler & Sunstein, 2008), and several scholars have claimed that choice architecture is unlikely to affect decisions when people have clear preferences (Johnson & Goldstein, 2003). To see how this assumption holds up in practice, we review empirical evidence on the potential moderating role of preexisting preferences with regard to nudge effectiveness.

Nudges have been proposed to “alter behavior in a predictable way” (Thaler & Sunstein, 2008) because people should all react similarly to certain adaptations in the choice architecture: On average, people are likely to stick to a selected default option because of the status-quo bias, and people are likely to overestimate the probability of events that received recent media coverage because of the availability bias (Tversky & Kahneman, 1974). However, accumulating evidence suggests that, despite the common cognitive biases that form the underlying mechanisms for many nudge interventions, people are nudgeable (i.e., show the “predictable response”) only if the promoted behavior aligns with their personal preferences. Two divergent situations will result in an ineffective nudge: People are not affected by a nudge when it promotes behavior that is not in line with their preferences or when they already have very strong preferences that are in line with the nudge. In the first case, people will ignore the nudge and select an alternative option; in the second case, people will perform the promoted behavior regardless of the presence of a nudge. For example, an opt-out default nudge that automatically directed part of people’s tax refunds into a savings account was found to be ineffective when people already had made plans to spend their refunds (Bronchetti et al., 2013). In another study, a nudge repositioning whole-wheat bread to a more convenient location in supermarket displays was ineffective, presumably because people just bought the bread they always bought (de Wijk et al., 2016). In these two examples, the role of preexisting preferences was inferred as a post hoc explanation for an ineffective nudge intervention. Other indirect evidence that a nudge does not overrule personal preferences comes from research showing that, when explicitly given the opportunity, only very few people choose to revise the choice they made when a default nudge was applied (Loewenstein et al., 2015).

A more explicit test of the moderating effect of personal preferences showed that the acceptance of “green” products promoted by a default nudge was higher for people with stronger proenvironmental attitudes (Taube & Vetter, 2019). At the same time, acceptance of a default-promoted nongreen option was lower among this group, suggesting that people with strong proenvironmental attitudes would still select the greener option regardless of the nudge. Another study found that participants’ choice for a small, medium, or large portion of soda was driven more strongly by their level of thirst (predicting relatively larger portion choices) and their level of health consciousness (predicting relatively smaller portion choices) regardless of the presence of a nudge (Venema et al., 2019). Illustrating the redundance of a nudge when participants already have very strong preferences for the nudged behavior, Theotokis & Manganari (2015) showed that the effect of a default nudge to promote towel reuse was much smaller for people who already were very environmentally conscious—they intended to reuse their towel anyway—than for people who were less concerned about the environment.

What we discussed so far applies to cases in which people’s preferences are clear. Often, however, preferences are ill-formed, or people may be ambivalent about their preferences. The question is how the absence of a clear preference for one specific option would affect nudgeability. It has been suggested that “inconsistent choosers” would be particularly susceptible to the influence of a nudge (Goldin, 2015), and more generally it has been shown that people more heavily rely on heuristics when they are uncertain about their decisions (Neth & Gigerenzer, 2015; Tversky & Kahneman, 1974). Only a few studies have explicitly addressed this question in the context of nudging, distinguishing two situations in which people have no clear preference: They can have conflicting goals, or they can just be indifferent. Both situations enhanced the effectiveness of a social-proof nudge, referring to the notion that the actions of other people are taken as “proof” that these actions must be correct (Cialdini, 1984): The nudge was more likely to guide people’s choices in a task they did not really care about, and in a different study people who experienced greater ambivalence toward a certain choice were more nudgeable than those who did have clearer preferences for either option (Venema et al., 2020). This finding aligns with research showing that people were less affected by a nudge when they were asked to first articulate their preferences—presumably yielding clearer preferences (Steffel et al., 2016).

Together, research so far corresponds to the idea that nudgeability as a function of preferences would best be captured by an inverted U shape such that people having strong preferences either against or in line with the nudge will be least affected (see Box 3). The current state of affairs does not allow strong conclusions on the existence of an inverted U shape but may serve as a hypothesis for modeling the association between preferences and effectiveness in future studies. This conclusion has imperative implications for the legitimacy of nudging. If it can be ensured that people will not be nudgeable when a promoted behavior is in conflict with their personal preference, concerns about nudging being a manipulative policy instrument are likely to be relieved. However, it should be emphasized that most studies on nudging so far have dealt with uncontroversial and simple behaviors such as eating healthily or reducing plastic waste, which are typical behaviors for which many people are at least somewhat motivated (Van Gestel et al., 2021; Venema et al., 2019). In view of the potential of nudges to engage people with pressing policy issues—such as poverty, early childhood development, productivity, or climate change (Organisation for Economic Co-operation and Development, 2017)—it is urgent to examine in what way people’s preferences can be taken into account when nudging for these bigger cases. It has been argued that nudges should focus more on social issues that transcend personal benefit (Van der Linden, 2018), but such an argument also calls for more systematic research into preferences when these kinds of complex social issues are at stake. This especially applies to themes that have generated heated societal debate, such as, for example, racism or vaccine hesitancy, which may be subject to strong political views. The extent to which choices relating to complex social issues are nudgeable should therefore be a prominent topic on the research agenda.

Box 3 **Inverted U Curve of Nudgeability According to Preferences**

The relation between individual preferences and the effectiveness of nudges seems best represented by an inverted U curve. People with less developed preferences (because they are ambivalent or in doubt about their choice) can be nudged toward a specific option. Those on the extreme left end, who have a clear preference for the alternative, will not be affected by the nudge. At the extreme right end of the inverted U shape we would find people who have strong preferences in line with the nudge; for this group nudges tend to be redundant because they would make the desired choice regardless of the presence of a nudge.

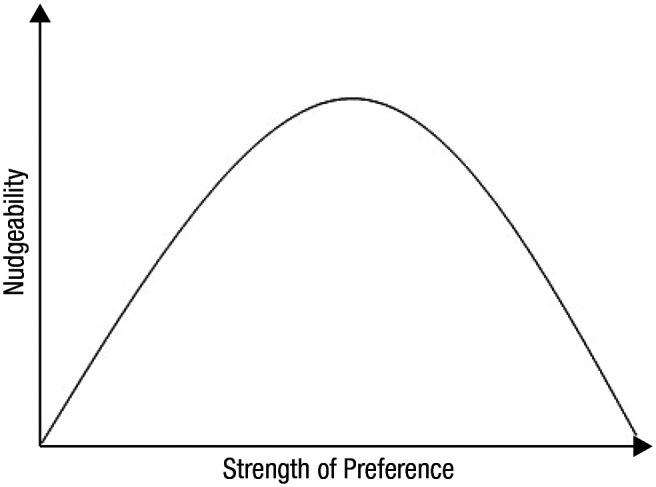

## Modus of Thinking

One of the most prevailing assumptions of nudge effectiveness is that they target System 1 processes (Marchiori et al., 2017; Marteau et al., 2012). That is, nudges are thought to strategically make use of cognitive biases and heuristics that regulate human behavior. Some researchers have even stated that nudges are supposed to “harness cognitive and motivational deficiencies” (Hertwig & Grüne-Yanoff, 2017, p. 974). Indeed, it has been argued that these cognitive “flaws” shape behavior in suboptimal ways (Evans & Stanovich, 2013), and the idea behind nudging techniques is to embrace these heuristics and biases by structuring the environment in such a way that it stimulates desirable outcomes. Consequently, it is often suggested that people should be more susceptible to nudges when they are in a System 1 mindset.

Empirical studies devoted to studying the effectiveness of nudges under System 1 processing have used a large heterogeneity of methods to install such a mindset, including measures and manipulations of self-control, cognitive load, and time pressure (see Box 1). However, most studies have found no evidence that nudges work best under System 1 conditions. In fact, most research has found that nudges result in similar levels of desirable behavior under System 1 conditions as under respective control conditions, resulting in main effects that were not moderated by System 1 mindsets. For example, research on the proximity effect—the effect that something is more likely to be chosen if it is placed closer by—has demonstrated that this effect is not affected by cognitive capacity (Hunter et al., 2018, 2019). Studies on the center-stage nudge (using the inclination to choose the middle option; Valenzuela & Raghubir, 2009) revealed a similar pattern; that is, the effect of the nudge did not increase when participants were in a state of low self-control (Missbach & König, 2016; Venema et al., 2019). In line with these findings, a study on increasing the effort to obtain snacks by means of sugar tongs revealed no moderation of cognitive load (Brunner, 2013). Taken together, these studies demonstrate that System 1 conditions do not enhance nudge effects compared with a control, as is often assumed.

Other research has demonstrated that nudges may buffer against suboptimal effects that would otherwise occur under System 1 mindsets. For example, in a study on food choices it was shown that hungry participants made fewer healthy choices than satiated participants when there was no nudge (i.e., social-proof information explaining that most people preferred the healthy food option), whereas there was no difference between satiated and hungry participants in the number of healthy choices when this nudge was present (Cheung et al., 2017). Likewise, it has been revealed that consumers low in self-control make less healthy choices than consumers high in self-control in the absence of a traffic-light label, whereas there was no difference between low and high self-control consumers when this nudge was present (Koenigstorfer et al., 2014). These studies seem to imply that nudges can be relatively more effective under System 1 mindsets, but in absolute terms they still do not demonstrate that nudgeability is enhanced under System 1.

Looking at the other side of the coin, there is little research on nudge effects under System 2 mindsets. Most studies have used manipulations to inhibit System 2 processing, and noninhibition has been used in control groups, but few studies have actually stimulated System 2 processing to examine whether nudge effects remain when people are better able to deliberate their decision. A recent investigation revealed that a default nudge stimulating sustainable amenities when moving to a new apartment (e.g., energy-efficient dishwasher or solar-powered outdoor lighting) remained effective when people were instructed to deliberate on their choice (Van Gestel et al., 2020). Simply having more time to choose does not inhibit default effects, but articulating preferences before choosing, and thus reasoning in a more balanced way, may render defaults less effective (Steffel et al., 2016). Likewise, prompting people to list positive aspects of the nonnudged option can eliminate default effects (Dinner et al., 2011). This finding shows that although it may not be simple to overcome default effects when people have the opportunity to consider their influence, it is also not impossible as long as people are willing and able to invest enough cognitive effort.

Taken together, current evidence does not support the assumption that nudge effects depend on System 1 processes. Most research suggests that nudges are equally effective under System 1 conditions compared with a control condition (of which it is unclear whether these control conditions truly entail System 2 processing); some studies indicate that people may have more to gain from a nudge while in a System 1 mindset. In terms of the legitimacy of nudging, the vast majority of studies indicate that nudges are not solely using System 1 mindsets such that people are steered in a direction to engage in behavior they would otherwise not perform. Concerns regarding the manipulative nature of nudges could possibly be lowered by the insight that people with lower cognitive capacity are not victims of nudge effects. If anything, most research suggests that nudges are equally effective across differing levels of cognitive resources. Moreover, if people possess the capacity and willingness to invest sufficient cognitive effort into making a balanced decision, current evidence suggests that people may deviate from typical nudge effects.

## Topics for the Nudgeability Research Agenda

In the previous sections, we emphasized the need for more research on the source of the nudge (e.g., government or private parties) and the subject of the nudge (simple behaviors vs. complex social issues) to examine whether these dimensions determine susceptibility to nudge influence. Another topic that warrants more investigation is whether poor living conditions are related to nudgeability. Compared with the previously discussed dimensions, there is only a very limited body of evidence that indicates whether and in what way being underprivileged is associated with being influenceable to nudges. Nevertheless, we find it important to review these findings in light of the discussion that has been raised about whether low SES may make people more responsive to nudges and, if so, whether people from underprivileged groups may benefit. It has been suggested that people specifically from groups with low income and/or education could benefit, as this kind of choice guidance does not rely on the comprehension of complex information required for making consequential decisions about health, finance, or energy (Marteau et al., 2012). Low income and/or education have been associated with cognitive scarcity (Mani et al., 2013), which may render the impact of educational-policy interventions ineffective because they depend on recipients’ literacy and numeracy. If people from disadvantaged groups could make better decisions in their own best (long-term) interest, this would contribute to a reduction in the welfare gap, and there would be marked socioeconomic differences in health and well-being. However, despite its importance for a fair public policy, there has been little systematic research on responsiveness to nudges in relation to the SES of target groups. Most nudging studies do not explicitly report on participants’ socioeconomic background, precluding an examination of how these characteristics affect susceptibility to nudge influence. Moreover, many nudge studies have been conducted in homogeneous samples of students or highly educated community residents (e.g., Loewenstein et al., 2015) who are not representative of the general population.

A handful of studies have suggested that people from different socioeconomic backgrounds respond equally to nudges (Hotard et al., 2019; Hunter et al., 2018; Johnson et al., 2002; Levy et al., 2012; Thunström, 2019). However, most studies have reported that people with low education and/or income may be more susceptible to nudge influence, although these studies are limited to default nudges with financial consequences, which may affect people with low income more than those who are financially better off. Typical examples are preselected opt-out rates for contributions to pension schemes and default green-energy contracts that are generally more expensive (Agnew & Szykman, 2005; Beshears et al., 2016; Ghesla et al., 2020; Madrian & Shea, 2001). The higher inclination to stick to the default among people with lower SES may have several reasons, such as being less informed about the topic of choice, perceiving the choice as more complex, seeing the default more often as expert recommendation, and having a higher tendency to put off making the choice because of decision inertia (Beshears et al., 2016; Ghesla et al., 2020). It has also been suggested that the greater effect of default nudges in people with low SES may relate to scarce cognitive resources (Bruns, 2019). There is, however, no evidence that cognitive scarcity leads to greater default effects, which challenges the role of low SES as a direct explanation of greater default susceptibility.

Higher responsiveness to nudges in people from disadvantaged groups may compensate for low literacy or decision fatigue in making complex choices. However, nudges may potentially increase the SES welfare gap because it disproportionately affects the poor in steering toward decisions they do not endorse. For instance, a field study on default choices in consumer electricity contracts led poorer households to pay more for their electricity consumption than they would have wanted to (Ghesla et al., 2020). However, another study examining the effects of a default manipulation in a sample of low-income tax filers showed that the nudge to save refunds was ineffective when people had made plans to spend the refund for consumption (Bronchetti et al., 2013).

Altogether, there is some evidence that people from lower SES backgrounds are more responsive to nudges, although studies primarily pertain to default nudges with financial consequences. Whether higher susceptibility to nudges is advantageous for specifically low-SES groups is as of yet uncertain, as mixed results have been reported on whether people can overrule the default when it does not match their preexisting preferences. As a result, specifically in disadvantaged groups more research is needed on other types of nudges as well as choices that relate to nonfinancial matters, such as healthy eating (Thunström, 2019).

## Discussion

In this article, we examined conditions that determine people’s susceptibility to nudge influence in an effort to probe common assumptions about when nudges are effective in guiding people’s choices. Although it has been repeatedly emphasized that nudges are “gentle directions” to promote decisions in people’s own best interest, there has been considerable debate about nudging legitimacy insofar as it may violate principles of good public policy that require transparency, acknowledgment of citizen preferences, and a reasonable degree of informed decision-making. We have highlighted these issues from the viewpoint of nudge effectiveness to determine nudgeability under conditions of disclosure of nudge presence and purpose, nudge-congruent and nudge-incongruent preferences, and either or not being able to deliberate on one’s choice (System 1 or System 2 processing).

Our review reveals that people are equally responsive to nudges regardless of whether their presence, purpose, or working mechanisms are disclosed—suggesting that transparency does not compromise nudge effects. Our analysis also shows that preexisting preferences matter insofar as nudges prove generally ineffective when not concordant with goals and intentions. Rather, nudges appear to have the greatest impact on choice when people have less developed preferences because they are ambivalent or in doubt about their choice. We further showed that nudges are not specifically effective when people are in a System 1 state of mind, which would, according to the prevailing assumption, make them more susceptible to nudge influence. It is uncertain, however, to what extent explicit encouragement to reflect on choices may attenuate nudge effects, although potentially weaker effects after a consideration of options may also be due to more articulated preferences.

Together, these findings call for greater scrutiny of the theoretical underpinnings of nudges. Nudges have been presented as typical System 1 devices targeting heuristics and biases that would require unawareness of their influence while, according to some (Bovens, 2009; Steffel et al., 2016), disregarding people’s preferences for a particular choice. More recent theoretical work displays growing attention for a new generation of nudges that explicitly target System 2 processes (Sunstein, 2016). “Overt” System 2 nudges that are easy to discern are generally better accepted by the target population (Bang et al., 2018; Felsen et al., 2013; Jung & Mellers, 2016; Sunstein, 2016), presumably because they do not rely on “unconscious processing.” Moreover, System 2 nudges are expected to boost people’s decision-making capacities (Hertwig, 2017; Hertwig & Grüne-Yanoff, 2017). Although these novel types of nudges may thus potentially be more (or at least equally) effective and more legitimate, our review shows that “traditional” nudges already do not depend on being hidden and operating in the dark.

In view of these findings, note that this initial review primarily serves the purpose of agenda setting. More systematic research on which conditions make people more (or less) responsive to nudges is warranted. This applies to all three dimensions of nudgeability that our central to our review. Once more evidence becomes available on these factors, a meta-analytic synthesis of the literature would give a deeper insight into how each of these factors determines nudgeability. Future research should also take into account what type of nudge is involved. As alluded to in the introduction, “nudge” is an umbrella term that relates to many types of interventions. A better categorization of nudges in general and a particular focus on the relevance of a distinction between so-called System 1 nudges (of which people are supposedly unaware) and System 2 nudges (that aim to support people in reflecting upon their choices) are needed to make a significant step forward in unraveling when and why people are susceptible to the influence of nudges.

Examining the role of the type of nudge is important because many studies on the conditions of nudge effectiveness have been conducted with defaults, which are considered to exert the strongest influence on choice and thus provide a critical test of nudgeability. However, even in view of the finding that default effects remain after disclosure and are weakened when they do not accord with preferences, concerns about the deceitful nature of defaults have persisted (Steffel et al., 2016). It is therefore urgent to systematically address softer categories of nudges such as repositioning, framing, and salience (Keller et al., 2011) and examine whether our observations on nudgeability apply to these milder classes of nudges.

In addition to a more systematic synthesis of research into nudgeability incorporating the type of nudge, a number of topics require more research to find out whether they affect susceptibility to nudge influence. This especially applies to the source of the nudge, the complexity of the issue of interest, as well as to the role of SES—topics we could touch on only briefly because of the lack of empirical evidence. In particular, nudgeability of disadvantaged groups is a topic that should be put high on the agenda for future research, as nudges are supposed to benefit the health, wealth, and happiness of all. Not much is yet known about the potential distributional consequences of nudges, as only a few studies have examined the extent to which nudges specifically would affect people from disadvantaged groups. Whereas some studies have suggested that people from low-SES groups are more responsive to default nudges (either or not to their own benefit), other studies have indicated that poor people benefit from nudges in a way that they would not have from conventional policy instruments, such as informational campaigns (Hotard et al., 2019).

Once again, we would like to emphasize that our review is a first attempt to document nudgeability with a specific focus on elements that have been generated in debates on nudging legitimacy. More research on other facets that are so far absent in studies on moderators of nudge effects is much needed for the systematic documentation of the conditions that make people responsive to nudges. An important candidate for further study is self-regulatory capacity, or the extent to which people can identify relevant goals and act on them. Theoretically, people with poor self-regulatory capacity might experience greater benefit from nudges, but as of yet it is unknown whether people with low competence to regulate their behavior are more or less susceptible to choice guidance. Initial research suggests that people with low trait self-control are somewhat less responsive to nudging than those who have high trait self-control (Thunström, 2019). However, whether people with poor self-regulatory skills are nudgeable may critically depend on self-insight; people who have less self-knowledge may be more opposed to nudges than those who have more self-knowledge. Social connectedness is another feature that may explain heterogeneous responses to nudges. Decision makers who have few social ties and only a few friends serve as potential role models for questions about important choices might be more susceptible to nudges that function as social cues when in doubt, as is also demonstrated in defaults serving as (implicit) recommendations for a certain choice.

Finally, returning to the debates on nudging legitimacy that we addressed at the beginning of this article, it seems that concerns should be softened insofar as nudges do impose choice without respecting basic ethical requirements for good public policy. More than a decade ago, philosopher Luc Bovens (2009) formulated the following four principles for nudging to be legitimate: A nudge should allow people to act in line with their overall preferences; a nudge should not induce a change in preferences that would not hold under nonnudge conditions; a nudge should not lead to “infantilization,” such that people are no longer capable of making autonomous decisions; and a nudge should be transparent so that people have control over being in a nudge situation. With the findings from our review in mind, it seems that these legitimacy requirements are fulfilled. Nudges do allow people to act in line with their overall preferences, nudges allow for making autonomous decisions insofar as nudge effects do not depend on being in a System 1 mode of thinking, and making the nudge transparent does not compromise nudge effects.

## Transparency

*Action Editor:* Laura A. King

*Editor:* Laura A. King
